# The association between gestational weight trajectories in women with gestational diabetes and their offspring's weight from birth to 40 months

**DOI:** 10.1186/s13098-023-01239-y

**Published:** 2024-01-13

**Authors:** Wei Zheng, Jia Wang, Yichen Li, Xiaorui Shang, Kaiwen Ma, Xianxian Yuan, Kexin Zhang, Ruihua Yang, Yuru Ma, Guanghui Li

**Affiliations:** 1 Division of Endocrinology and Metabolism, Department of Obstetrics, Beijing Obstetrics and Gynecology Hospital, Capital Medical University, No 251, Yaojiayuan Road, Chaoyang District, Beijing, 100026 China; 2 Beijing Maternal and Child Health Care Hospital, Beijing, 100026 China; 3 Department of Children’s Health Care, Beijing Obstetrics and Gynecology Hospital, Capital Medical University, Beijing, 100026 China

**Keywords:** Gestational weight gain, Gestational diabetes mellitus, Macrosomia, Large for gestational diabetes, Childhood obesity, Trajectory

## Abstract

**Aims:**

To identify the gestational weight gain (GWG) patterns in women with gestational diabetes mellitus (GDM) and evaluate their association with offspring weight status from birth to 40 months.

**Materials and methods:**

This study included 2,723 GDM-mother–child pairs from the Beijing Birth Cohort Study. The association between GWG trajectories identified by the latent class model and offspring weight outcomes from birth to 40 months were evaluated, after adjustment for maternal age, parity, pre-pregnancy body mass index, maternal height, and blood glucose levels.

**Results:**

Three GWG rate groups, including the non-excessive GWG group (1,994/2,732), excessive GWG group (598 /2,732), and excessive early GWG group (140/2,732), were identified in women with GDM, respectively. Compared to the non-excessive GWG group, the adjusted OR (aOR) and 95% CI were 1.83 (1.35–2.47) and 1.79 (1.06–3.01) for macrosomia, 1.33 (1.07–1.66) and 1.48 (1.01–2.17) for large for gestational age (LGA) in the excessive GWG group and excessive early GWG group. Excessive GWG was also associated with an increased risk of BMI-for-age at 40 months (aOR = 1.66, 95% CI 1.14–2.42).

**Conclusions:**

Both excessive GWG and excessive early GWG increased the risk of macrosomia and LGA in women with GDM, but only the excessive GWG was associated with childhood overweight/obesity. The results suggest the long-term impact of GWG on offspring weight status in women with GDM and the potential benefits of GWG restriction after GDM diagnosis.

**Supplementary Information:**

The online version contains supplementary material available at 10.1186/s13098-023-01239-y.

## Introduction

Gestational diabetes mellitus (GDM) is a widely prevalent complication during pregnancy, affecting approximately one in six live births globally, with 84% of cases being GDM [1]. This condition poses a significant clinical risk to both maternal and offspring health, including a heightened risk of pregnancy complications, cesarean section, preterm delivery, and risks of large babies [2]. The most common health consequences observed in offspring are macrosomia and large for gestational age (LGA) [3]. Furthermore, recent studies have demonstrated a link between GDM and an increased risk of childhood obesity in offspring [4]. Some research has also suggested that this association may be influenced by maternal weight [5–7].

GDM, maternal obesity, and excess gestational weight gain (GWG) are interrelated risk factors for unfavorable outcomes in offspring. Excessive body weight is a significant modifiable risk factor for GDM, which is also associated with elevated risks of adverse outcomes for both mother and child [8, 9]. Studies have indicated that approximately one-third of the risk of delivering a large-for-gestational-age (LGA) infant is attributable to excessive GWG, while pre-pregnancy obesity can contribute to up to 21.6% of childhood overweight/obesity [10, 11]. This underscores the importance of managing GWG prior to and during pregnancy. Given the combined effects of GDM and excessive body weight on the risk of offspring overweight and obesity, researchers have sought to examine the relationship between GWG and pregnancy outcomes in women with GDM [12–14]. A meta-analysis by Viecceli et al. demonstrated that excessive GWG is a risk factor for adverse pregnancy outcomes, whereas restricted GWG can serve as a protective measure against macrosomia and LGA in women with GDM [12].

The impact of gestational weight gain (GWG) on the health outcomes of offspring may vary depending on the stage of pregnancy [13, 15, 16]. Pregnant women can achieve comparable GWG levels through different weight trajectories. Specifically, women with gestational diabetes mellitus (GDM) may exhibit a distinct weight gain pattern due to stringent weight management after GDM diagnosis. However, previous studies have primarily focused on total GWG or GWG within specific trimesters, with limited evidence on the association between maternal weight gain patterns throughout pregnancy and neonatal birth weight in women with GDM.

Therefore, this study aimed to identify potential GWG trajectory patterns in women with GDM and assess their associations with longitudinal changes in offspring anthropometric outcomes.

## Methods and materials

### Study design and participants

The study cohort consisted of singleton pregnant women diagnosed with GDM and their offspring, who were derived from the Beijing Birth Cohort Study. Eligible participants were Han ethnic group women aged 18–45 years, enrolled in the original cohort between 8 and 12 weeks of gestation, and expected to give birth at the Beijing Obstetrics and Gynecology Hospital, Capital Medical University between January 2014 and December 2017. Women with pre-existing chronic conditions such as diabetes and hypertension were excluded from the study. Specifically, we focused on recruiting women diagnosed with GDM. Those without GDM were excluded based on the 75 g oral glucose tolerance test (OGTT) conducted during 24–28 weeks of gestation. Additionally, participants lacking baseline information or maternal/offspring follow-up data were also excluded from the study. Further details on the inclusion and exclusion criteria, as well as the number of participants recruited, screened, and included in the analysis, can be found in Additional file 1: Figure S1. This study received approval from the Ethics Committee of the Beijing Obstetrics and Gynecology Hospital (2018-KY-009-01), and written informed consent was obtained from all participants.

### Measurements and outcomes

All participants were followed up in the hospital outpatient every month in early and mid-pregnancy and every 2 weeks from late pregnancy until delivery. The offspring of the women with GDM were followed from birth until their entrance into kindergarten at 3–4 years of age. Follow-up assessments of the offspring were conducted at birth, 5–6 months of age, 8–9 months of age, 11–12 months of age, 18 months of age, 24 months of age, and 40 months of age.

Trained research staff collected baseline information through a questionnaire, while perinatal outcomes were obtained from medical records. Anthropometric measurements for both maternal and offspring were taken by trained medical staff. These measurements were conducted in the outpatient department of the Beijing Obstetrics and Gynecology Hospital for the mothers and in the primary child healthcare settings for the offspring. However, the pre-pregnancy weight was self-reported by the participants.

Participants with pre-pregnancy body mass index (BMI) < 18.5 kg/m^2^, 18.5–23.9 kg/m^2^, 24–27.9 kg/m^2^, and ≥ 28 kg/m^2^ were classified as underweight, normal weight, overweight, and obesity according to guidelines for the prevention and control of overweight and obesity in Chinese adults [17]. The recommended total gestational weight gain (GWG) and weight gain rate were 11–16 kg, 8–14 kg, 7–11 kg, and 5–9 kg, and 0.46 kg/week, 0.37 kg/week, 0.30 kg/week, and 0.22 kg/week, respectively, for women with underweight, normal weight, overweight, and obesity, according to standard of recommendation for weight gain during pregnancy period (WS/T 801-2022).

Participants without preexisting diabetes went through a 75 g OGTT at 24–28 weeks of gestational age according to guidelines for the management of gestational diabetes mellitus [18]. Women with fasting blood glucose ≥ 5.1 mmol/L, 1 h blood glucose ≥ 10.0 mmol/L, or 2 h blood glucose ≥ 8.5 mmol/L were diagnosed with GDM [18].

The neonatal outcomes in this study were macrosomia, LGA, low birth weight (LBW), SGA, preterm delivery, and birth by cesarean section. Neonatal birth weight was defined as follows: Macrosomia: > 4,000 g, LBW: < 2,500 g, LGA: above the 90th percentile for gestational age, SGA: below the 10th percentile for gestational age. The cut-off value for LGA and SGA were from the international standards for newborn weight by Villar et al. [19]. Preterm delivery refers to delivery between 28 and 36 weeks of gestation. Offspring outcomes, including weight-for-length (WFL) from 6 ~ 18 months and BMI-for-age from 24 ~ 40 months, was calculated according to the World Health Organization Child Growth Standards [20]. BM- for-age >  + 2SD or < -2SD was classified as childhood overweight/obesity or thinness.

### Statistical analysis

The gestational weight gain rates of the participants were calculated by dividing the weight gain between two follow-up visits by the number of gestational weeks between those visits. To examine potential gestational weight gain patterns, the ratio of actual weight gain rates to recommended weight gain rates was utilized in a latent class trajectory analysis. The optimal number of trajectory groups was identified based on the lowest ΔBayesian information criteria (BIC) value. The Wilcoxon or chi-square test was employed to compare the baseline characteristics of participants across different gestational weight gain trajectories.

In this study, we constructed two-level fixed effect multilevel models to assess the dynamics of offspring weight status across various maternal gestational weight gain (GWG) groups. The first level represented repeated weight status measurements that were clustered within individual offspring, whereas the second level represented the individual level. Additionally, we incorporated interaction terms to estimate the difference in the change in the offspring's anthropometrics between different groups. Subsequently, we evaluated the association between GWG trajectory groups and the risk of categorical adverse pregnancy outcomes, including macrosomia, LGA, LBW, SGA, preterm delivery, birth by cesarean section, and BMI-for-age at 40 months >  + 2SD or < −2SD.

Moreover, ORs were calculated to evaluate the combined effect of pre-pregnancy BMI and weight gain trajectories during pregnancy, and the effect of GWG trajectories stratified by pre-pregnancy BMI. The above models were adjusted for maternal age, parity, pre-pregnancy BMI, maternal height, and blood glucose levels during OGTT. All statistical analyses were conducted using SAS 9.4.

## Results

### Three GWG rate patterns identified in women with GDM

**Fig. 1:**
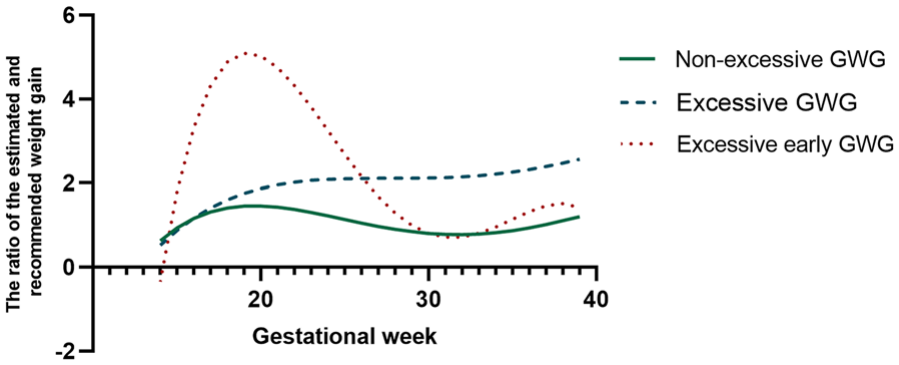
GWG rate patterns identified by latent class trajectory models

A total of 2,732 participants with GDM and their offspring were included in the analyses. Three GWG rate patterns were identified by latent class trajectory models (Fig. 1). Among the participants, the majority (72.9%) belong to the non-excessive GWG group (1,994/2,732), while 21.9% were classified as the excessive GWG group (598 /2,732), and only 5.1% assigned to the excessive early GWG group (140/2,732). Women with non-excessive GWG showed the lowest total GWG and the lowest GWG rate before the OGTT. On the contrary, women who experienced excessive early GWG showed the highest rate of GWG before the OGTT, and a restricted rate of GWG in late pregnancy.

Women who experienced excessive GWG showed a consistently elevated GWG rate throughout pregnancy compared to those in the non-excessive GWG group (as shown in Table 1). Additionally, maternal age, pre-pregnancy body mass index (BMI), and blood glucose levels varied significantly between the three GWG groups (Table 1). The participants with excessive GWG and excessive early GWG exhibited a higher percentage of pre-pregnancy overweight or obese, according to Table 1.

**Table 1:** Characteristics of the women with GDM stratified by GWG trajectory groups (p50(p25-p75) or n(%))

	Non-excessive GWG	Excessive GWG	Excessive early GWG	p-value†
N	1994	598	140	
Maternal age, year	32 (29–34)	31 (29–34)	31 (29–34)	0.0004
Parity				0.95
1st	1560 (78.23)	468 (78.26)	108 (77.14)	
2nd or more	434 (21.77)	130 (21.74)	32 (22.86)	
Height, cm	162 (160–165)	163 (160–167)	162 (160–165)	< 0.0001
Pre-pregnancy weight, kg	58 (52–65)	63 (56–72)	62 (55–70)	< 0.0001
Pre-pregnancy BMI, kg/m^2^	21.9 (19.9–24.4)	24.0 (20.9–27.2)	24.0 (21.4–26.4)	< 0.0001
Proportions by pre-pregnancy BMI category				< 0.0001
Underweight	221 (11.08)	14 (2.34)	3 (2.14)	
Normal weight	1220 (61.18)	277 (46.32)	65 (46.43)	
Overweight	386 (19.36)	180 (30.10)	45 (32.14)	
Obesity	167 (8.38)	127 (21.24)	27 (19.29)	
Weight at delivery, kg	70.0 (65.0–77.0)	81.0 (73.0–89.0)	78.0 (70.6–86.0)	< 0.0001
Total GWG, kg	12.0 (9.0–15.0)	17.0 (13.0–20.0)	14.5 (11.0–18.0)	< 0.0001
GWG before OGTT, kg	8.0 (5.5–10.0)	8.0 (5.5–11.0)	10 (7.5–13.0)	< 0.0001
GWG after OGTT, kg	4.0 (2.6–6.0)	8.0 (6.5–10.0)	4.5 (3.0–6.0)	< 0.0001
Gestational week of OGTT, week	25 (24–26)	25 (24–26)	25 (24–26)	0.06
Blood glucose levels, mmol/L				
OGTT 0 h	5.11 (4.65–5.33)	5.17 (4.82–5.38)	5.23 (5.01–5.54)	< 0.0001
OGTT 1 h	10.02 (8.81–10.7)	9.39 (7.95–10.36)	9.94 (8.50–10.95)	< 0.0001
OGTT 2 h	8.35 (7.15–9.09)	7.56 (6.57–8.70)	7.85 (6.63–8.88)	< 0.0001

*GDM* gestational diabetes mellitus, *GWG* gestational weight gain, *BMI* body mass index, *OGTT* oral glucose tolerance test

^†^p-values were calculated by the Wilcoxon test or chi-square test

### Excessive GWG is a risk factor for adverse perinatal and offspring weight dynamics from birth to 40 months

We compared the longitudinal dynamics of the offspring weight status from birth to 40 months of age among three groups with different GWG trajectories by two-level multilevel analysis for repeated measurements. Our findings revealed significant differences in weight status among the three groups, with the group category, age category, and interaction term showing statistical significance (p = 0.04), as indicated in Table 2.

**Table 2:** Comparison of longitudinal dynamics of the offspring weight status between GWG trajectory groups by multilevel analysis for repeated measurements (p50(p25-p75))

	Non-excessive GWG	Excessive GWG	Excessive early GWG	p-value†
Gestational age, week	39 (38–39)	39 (38–39)	39 (38–39)	0.15
Weight measurements				0.04
Neonatal birth weight, g	3385 (3093–3660)	3510 (3180–3810)	3488 (3245–3750)	
WFL at 5 ~ 6 months	−0.12 (−0.81 to 0.66)	0.05 (−0.79 to 0.8)	0.05 (−0.91 to 0.80)	
WFL at 8 ~ 9 months	0.05 (−0.66 to 0.61)	0.18 (−0.50 to 0.80)	0.18 (−0.39 to 0.61)	
WFL at 11 ~ 12 months	0.05 (−0.66 to 0.61)	0.13 (−0.52 to 0.76)	0.13 (−0.41 to 0.76)	
WFL at 18 months	0.01 (−0.73 to 0.52)	0.03 (−0.53 to 0.73)	0.01 (−0.55 to 0.74)	
BMI-for-age at 24 months	−0.25 (−0.92 to 0.39)	−0.09 (−0.72 to 0.46)	0.02 (−0.39 to 0.73)	
BMI-for-age at 40 months	−0.29 (−0.93 to 0.47)	−0.03 (−0.65 to 0.73)	−0.06 (−0.56 to 0.79)	

*WFL* weight-for-length, *BMI* body mass index

^†^The models were adjusted for maternal age, parity, pre-pregnancy BMI, maternal height, and blood glucose levels during OGTT

Furthermore, we investigated the association between different GWG groups and categorical adverse pregnancy outcomes. After controlling for confounding factors, the excessive GWG group and excessive early GWG group demonstrated higher risks for macrosomia and LGA compared to women with non-excessive GWG, as shown in Table 3. Excessive GWG was also associated with an increased risk of cesarean section and BMI-for-age at 40 months >  + 2SD compared to the non-excessive GWG group, after adjusting for potential confounders. However, excessive early GWG did not show a significant association with these outcomes.

**Table 3:** Association between GWG trajectory groups and risk of adverse perinatal and offspring outcomes at 40 months

	Crude OR	Adjusted OR1†	Adjusted OR2‡
Macrosomia			
Non-excessive GWG	Ref	Ref	Ref
Excessive GWG	2.62 (1.99–3.47)	2.01 (1.51–2.69)	1.83 (1.35–2.47)
Excessive early GWG	2.25 (1.37–3.74)	1.84 (1.10–3.07)	1.79 (1.06–3.01)
LGA			
Non-excessive GWG	Ref	Ref	Ref
Excessive GWG	1.77 (1.44–2.16)	1.45 (1.17–1.79)	1.33 (1.07–1.66)
Excessive early GWG	1.78 (1.24–2.57)	1.51 (1.04–2.20)	1.48 (1.01–2.17)
LBW			
Non-excessive GWG	Ref	Ref	Ref
Excessive GWG	1.02 (0.62–1.67)	1.03 (0.62–1.71)	1.15 (0.67–1.95)
Excessive early GWG	0.20 (0.03–1.46)	0.21 (0.03–1.49)	0.22 (0.03–1.61)
SGA			
Non-excessive GWG	Ref	Ref	Ref
Excessive GWG	0.54 (0.24–1.20)	0.60 (0.26–1.35)	0.70 (0.31–1.62)
Excessive early GWG	0.65 (0.16–2.71)	0.72 (0.17–3.01)	0.81 (0.19–3.42)
Preterm			
Non-excessive GWG	Ref	Ref	Ref
Excessive GWG	1.41 (0.96–2.07)	1.38 (0.93–2.05)	1.54 (1.02–2.33)
Excessive early GWG	0.75 (0.30–1.87)	0.74 (0.30–1.87)	0.78 (0.31–1.97)
Cesarean section			
Non-excessive GWG	Ref	Ref	Ref
Excessive GWG	1.41 (1.17–1.70)	1.46 (1.18–1.79)	1.64 (1.32–2.03)
Excessive early GWG	1.37 (0.96–1.94)	1.43 (0.98–2.09)	1.42 (0.96–2.09)
BMI-for-age at 40 months > + 2SD			
Non-excessive GWG	Ref	Ref	Ref
Excessive GWG	1.99 (1.40–2.84)	1.66 (1.15–2.41)	1.66 (1.14–2.42)
Excessive early GWG	1.31 (0.62–2.77)	1.14 (0.53–2.43)	1.13 (0.53–2.42)
BMI-for-age at 40 months < -2SD			
Non-excessive GWG	Ref	Ref	Ref
Excessive GWG	0.63 (0.34–1.18)	0.75 (0.40–1.42)	0.79 (0.42–1.52)
Excessive early GWG	0.23 (0.03–1.68)	0.27 (0.04–1.94)	0.26 (0.04–1.91)

*GWG* gestational weight gain, *LGA* large for gestational age, *LBW* low birth weight, *SGA* small for gestational age, *WFA* weight for age, *BMI* body mass index

^†^Models were adjusted for maternal age, parity, and pre-pregnancy BMI

^‡^Models evaluating risk for perinatal outcomes were adjusted for maternal age, parity, pre-pregnancy BMI, maternal height, and blood glucose levels during OGTT

### Maternal overweight/obesity modified the effect of excessive GWG on adverse perinatal and offspring outcomes at 40 months

We further evaluate the joint effects of pre-pregnancy overweight/obesity and GWG patterns on the development of macrosomia and LGA in offspring. The results from Table 4 indicate that both pre-pregnancy overweight/obesity and excessive GWG during pregnancy were found to be significant risk factors for macrosomia, LGA, and delivery via cesarean section. Moreover, the risk further escalated when both factors coexisted. Additionally, the combination of pre-pregnancy overweight/obesity and excessive GWG during pregnancy was also associated with adverse offspring BMI-for-age at 40 months. However, excessive early GWG did not show any significant association with adverse perinatal or offspring BMI-for-age at 40 months, as illustrated in Table 4.

**Table 4:** Combined effect of pre-pregnancy BMI and GWG trajectory on the risk of adverse offspring outcomes

Pre-pregnancy BMI	GWG trajectory groups	Crude OR	Adjusted OR1†	Adjusted OR2‡
Macrosomia				
Underweight or Normal weight	Non-excessive GWG	Ref	Ref	Ref
Underweight or Normal weight	Excessive GWG	2.53 (1.65–3.86)	2.48 (1.62–3.80)	2.20 (1.42–3.40)
Underweight or Normal weight	Excessive early GWG	1.47 (0.57–3.75)	1.43 (0.56–3.67)	1.36 (0.53–3.52)
Overweight/Obese	Non-excessive GWG	2.38 (1.67–3.38)	2.40 (1.68–3.41)	2.25 (1.56–3.23)
Overweight/Obese	Excessive GWG	4.68 (3.25–6.73)	4.62 (3.21–6.65)	3.89 (2.67–5.67)
Overweight/Obese	Excessive early GWG	4.86 (2.63–8.99)	4.80 (2.59–8.89)	4.37 (2.33–8.20)
LGA				
Underweight or Normal weight	Non-excessive GWG	Ref	Ref	Ref
Underweight or Normal weight	Excessive GWG	1.44 (1.07–1.93)	1.42 (1.06–1.91)	1.31 (0.96–1.77)
Underweight or Normal weight	Excessive early GWG	1.75 (1.02–2.99)	1.74 (1.02–2.99)	1.69 (0.98–2.91)
Overweight/Obese	Non-excessive GWG	1.75 (1.40–2.20)	1.68 (1.33–2.11)	1.61 (1.27–2.04)
Overweight/Obese	Excessive GWG	2.90 (2.22–3.77)	2.86 (2.19–3.72)	2.51 (1.91–3.29)
Overweight/Obese	Excessive early GWG	2.51 (1.53–4.12)	2.44 (1.48–4.01)	2.26 (1.36–3.74)
Cesarean section				
Underweight or Normal weight	Non-excessive GWG	Ref	Ref	Ref
Underweight or Normal weight	Excessive GWG	1.50 (1.16–1.95)	1.73 (1.31–2.29)	1.97 (1.47–2.63)
Underweight or Normal weight	Excessive early GWG	1.15 (0.69–1.94)	1.36 (0.78–2.36)	1.38 (0.79–2.43)
Overweight/Obese	Non-excessive GWG	1.90 (1.55–2.33)	1.65 (1.33–2.05)	1.56 (1.25–1.95)
Overweight/Obese	Excessive GWG	1.91 (1.49–2.46)	2.13 (1.62–2.80)	2.20 (1.66–2.91)
Overweight/Obese	Excessive early GWG	2.28 (1.42–3.67)	2.44 (1.47–4.05)	2.20 (1.30–3.72)
BMI-for-age at 40 months > + 2SD				
Underweight or Normal weight	Non-excessive GWG	Ref	Ref	Ref
Underweight or Normal weight	Excessive GWG	1.45 (0.84–2.50)	1.43 (0.83–2.47)	1.38 (0.80–2.40)
Underweight or Normal weight	Excessive early GWG	1.04 (0.32–3.44)	1.01 (0.31–3.33)	0.96 (0.29–3.19)
Overweight/Obese	Non-excessive GWG	1.19 (0.76–1.87)	1.28 (0.81–2.02)	1.18 (0.74–1.89)
Overweight/Obese	Excessive GWG	2.74 (1.76–4.26)	2.77 (1.78–4.31)	2.64 (1.69–4.13)
Overweight/Obese	Excessive early GWG	1.71 (0.66–4.42)	1.75 (0.67–4.54)	1.66 (0.63–4.34)

*BMI* body mass index, *GWG* gestational weight gain, *LGA* large for gestational age, *WFA* weight for age

^†^Models were adjusted for maternal age and parity

^‡^Models evaluating risk for perinatal outcomes were adjusted for maternal age, parity, maternal height, and blood glucose levels during OGTT

According to results from the analysis stratified by pre-pregnancy BMI status, excessive GWG heightened the risk of macrosomia/LGA and BMI-for-age at 40 months in women with overweight/obesity. Conversely, in the underweight or normal weight subgroup, excessive GWG amplified the probability of macrosomia and cesarean delivery. However, no substantial correlation between excessive early GWG and unfavorable offspring outcomes was detected in either cohort (Additional file 1: Figure S2).

## Discussion

Based on the findings of this study, three potential patterns of GWG were identified by a latent class model, namely non-excessive GWG, excessive GWG, and excessive early GWG, in women diagnosed with GDM. The excessive GWG and excessive early GWG groups were observed to have increased risks of having babies with macrosomia and LGA compared to the non-excessive GWG group. Furthermore, excessive GWG, but not excessive early GWG, was found to be associated with childhood overweight/obesity in offspring from birth to 3–4 years of age. The combined effect of pre-pregnancy overweight/obesity and excessive GWG pattern was also shown to increase the risk of macrosomia, LGA, and childhood overweight/obesity at 40 months of age.

There has been considerable evidence linking excessive GWG to an increased risk of LGA infants, macrosomia, and childhood obesity [8, 11]. However, most previous studies have focused on total GWG or GWG during specific periods during pregnancy, limiting the ability to comprehensively capture GWG dynamics throughout pregnancy [8, 11]. This limitation is particularly relevant for women with GDM, as they may exhibit distinct GWG patterns before and after GDM diagnosis [13, 21, 22].

To our best knowledge, this is the first study to recognize GWG patterns throughout pregnancy in women with GDM. The use of latent class trajectory analysis has been instrumental in characterizing weight gain patterns during pregnancy and exploring their association with birth and childhood outcomes in recent studies [23–25]. For instance, Pugh et al. identified four GWG trajectories that were associated with neonatal birth weight [23], while our previous study recognized four GWG trajectories in women with overweight/obesity and demonstrated that elevated GWG in early and mid-pregnancy was a risk factor for LGA infants [24]. A study by Xu et al. showed that a high-stable increasing pattern of the GWG during pregnancy is a risk factor for overweight/obesity in offspring at 3 years of age [25]. These findings highlight the advantages of the trajectory approach in comprehensively evaluating the association between maternal weight and offspring growth.

Based on our earlier research and previous reports, it has been observed women may exhibit different weight gain patterns both before and after being diagnosis with GDM [13, 21, 22]. The diagnostic OGTT for GDM is typically performed between the 24th and 28th week of gestation, when the physiological insulin resistance is established [26]. A considerable proportion of women with GDM may have already gained excessive weight by the time of diagnosis [27]. However, it is important to note that some of these women may conscientiously manage their GWG after being diagnosed with GDM.

Analyzing the isolated GWG during a specific period of pregnancy alone may not provide a comprehensive evaluation of the impact of maternal weight changes on the weight of the offspring. Therefore, we employed the latent class trajectory technique to identify specific GWG patterns in women diagnosed with GDM and investigate their associations with pregnancy outcomes. This approach has been proven effective in identifying distinct types of GWG trajectories, considering the unique characteristics of weight gain in women with GDM.

Based on our analysis, we identified three distinct types of GWG trajectories. The majority of women exhibited non-excessive GWG patterns, while the remaining participants were classified into two groups based on their weight control status before and after the diagnosis of GDM: excessive GWG or excessive early GWG patterns.

This study provides evidence that both excessive GWG and excessive early GWG in women with GDM are associated with an increased risk of macrosomia and LGA. However, only excessive GWG throughout pregnancy was found to be linked to childhood overweight/obesity at 40 months of age. These findings indicate that managing GWG after GDM diagnosis may help reduce the risk of offspring overweight/obesity. Consistent with our results, recent studies also demonstrated a positive association between GWG following GDM diagnosis and the risk of LGA and macrosomia [21, 22]. Previous evidence also suggested that controlling GWG after GDM diagnosis may be advantageous for preventing adverse pregnancy outcomes [13, 28, 29]. In this study, we further identified the potential benefits of limiting GWG after GDM diagnosis for long-term weight control in offspring.

Clinicians may have reservations about recommending weight control during GDM management due to concerns about potentially increasing the rate of delivering small for gestational age (SGA) infants. However, our study did not find a significant difference in the rate of delivering SGA or low birth weight (LBW) infants among individuals with different patterns of GWG. This finding is consistent with previous research indicating that weight control during GDM management in individuals with excessive early GWG does not increase the risk of SGA [13, 28, 29].

Accumulating evidence supports the notion that both pre-pregnancy obesity and excessive GWG during pregnancy are significant risk factors for childhood obesity [10, 11]. Our findings further revealed that the combined effect of pre-pregnancy overweight/obesity and excessive GWG was linked to higher birth weight and an increased risk of childhood obesity. Additionally, this study demonstrated that the impact of excessive GWG on offspring outcomes in women with GDM is modified by pre-pregnancy overweight/obesity. Specifically, excessive GWG was found to be associated with higher BMI-for-age at 40 months of age in women with overweight and obesity, but not in underweight/normal weight women. Moreover, excessive early GWG did not increase the risk of childhood obesity in either the underweight/normal weight or the overweight and obese group. These results underscore the importance of addressing both pre-pregnancy and gestational weight management in preventing childhood obesity, particularly among high-risk populations such as women with GDM.

The presence of hyperglycemia itself poses a well-established risk for macrosomia and LGA [30]. Maternal glucose serves as the primary energy source for fetal growth. Maternal hyperglycemia increases the transfer of glucose, amino acids, and free fatty acids across the placenta. This leads to fetal overnutrition and hyperinsulinemia, contributing to the development of obesity in offspring [31]. Furthermore, the abnormal intrauterine environment resulting from maternal hyperglycemia can have long-term effects on the health of the offspring through epigenetic modifications [32, 33].

Therefore, when women with GDM experience excessive weight gain during pregnancy, the risk of offspring obesity is further amplified. Effective weight management for GDM patients becomes even more crucial compared to non-diabetic pregnant women, especially for those who have already surpassed the optimal weight gain targets at the time of GDM diagnosis. Our study findings suggest that there is still an opportunity to positively manage weight after GDM diagnosis to improve offspring weight status. Moreover, our results underscore the need for stronger support in achieving healthy maternal weight gain both before and during GDM management. Addressing excessive weight gain during pregnancy in GDM patients can have multiple potential benefits, including reducing the risk of adverse perinatal outcomes and mitigating the long-term consequences of offspring obesity.

The study has certain limitations that need be acknowledged. Firstly, weight information was collected monthly during mid-pregnancy and twice a week in late pregnancy. However, only one weight record between 13 and 16 weeks of gestation was gathered to calculated the average GWG rate in early pregnancy, which impeded the depiction of the early GWG curve. Secondly, the sample size was relatively small for the excessive early GWG group identified by the latent class model. The absence of significant differences in the results may be attributed to the limited sample size of the excessive early GWG group. Thirdly, potential confounding factors such as diet pattern and physical activity of the offspring were not available in this study. The lack of the related information of offspring may limit the comprehensive understanding of the relationship between maternal GWG during pregnancy and offspring weight status.

Despite these limitations, our study provides valuable insights into the long-term impact of excessive weight gain during pregnancy on offspring health in women with GDM. These findings underscore the significance of weight management in GDM patients, emphasizing the importance of early intervention and continuous management of GWG throughout pregnancy. Additionally, our results suggest that restricting GWG after GDM diagnosis also holds potential benefits. Overall, GWG management both before and after GDM diagnosis may have the potential to improve the weight status of future generations.

### Supplementary Information

**Additional file 1: Figure S1.** Flow chart of the selection of study participants.

**Additional file 2: Figure S2.** Adjusted OR for the adverse offspring outcomes in women with different GWG trajectories stratified by body mass index status. OR was adjusted for maternal age, parity, pre-pregnancy BMI, maternal height, and blood glucose levels during OGTT. OR for WFA outcomes at 40 months were additionally adjusted for offspring height at 40 months. * indicated significant difference (p < 0.05) compared to the non-excessive GWG group.

## Data Availability

The datasets and code used and/or analyzed during the current study are available from the corresponding author on reasonable request.

## Abbreviations

GDM: Gestational diabetes mellitus
GWG: Gestational weight gain
LGA: Large for gestational age
OGTT: Oral glucose tolerance test
BMI: Body mass index
BIC: Bayesian information criteria
