# Supplementary material for: The association between gestational weight trajectories in women with gestational diabetes and their offspring's weight from birth to 40 months
Source: Diabetol Metab Syndr. 2024 Jan 13;16:17. doi: 10.1186/s13098-023-01239-y (PMC10790252; doi:10.1186/s13098-023-01239-y)
Supplement: Supplementary file 1 — Additional file 1: Figure S1. Flow chart of the selection of study participants. [file 13098_2023_1239_MOESM1_ESM.docx]

Figure S1 Flow chart of the selection of study participants

Women from the original cohort who were pregnant gave birth in this hospital between January 2014 and December 2017 (n=21, 075)

**Inclusion criteria:**

- Women with gestational diabetes mellitus
- Singleton pregnancy
- Enrollment age 18-45 years old

Eligible participants

(n=3,259)

Included in the final analysis

(n=2, 732)

**Exclusion criteria:**

- Nine diabetes or hypertension before pregnancy
- 23 stillbirth or miscarriage
- Two lack of baseline information
- 67 maternal weight or height were missing or outlier
- 460 without offspring anthropometrics
