# Supplementary figures and images for: The association between gestational weight trajectories in women with gestational diabetes and their offspring's weight from birth to 40 months
Source: Diabetol Metab Syndr. 2024 Jan 13;16:17. doi: 10.1186/s13098-023-01239-y (PMC10790252; doi:10.1186/s13098-023-01239-y)

Figure S2

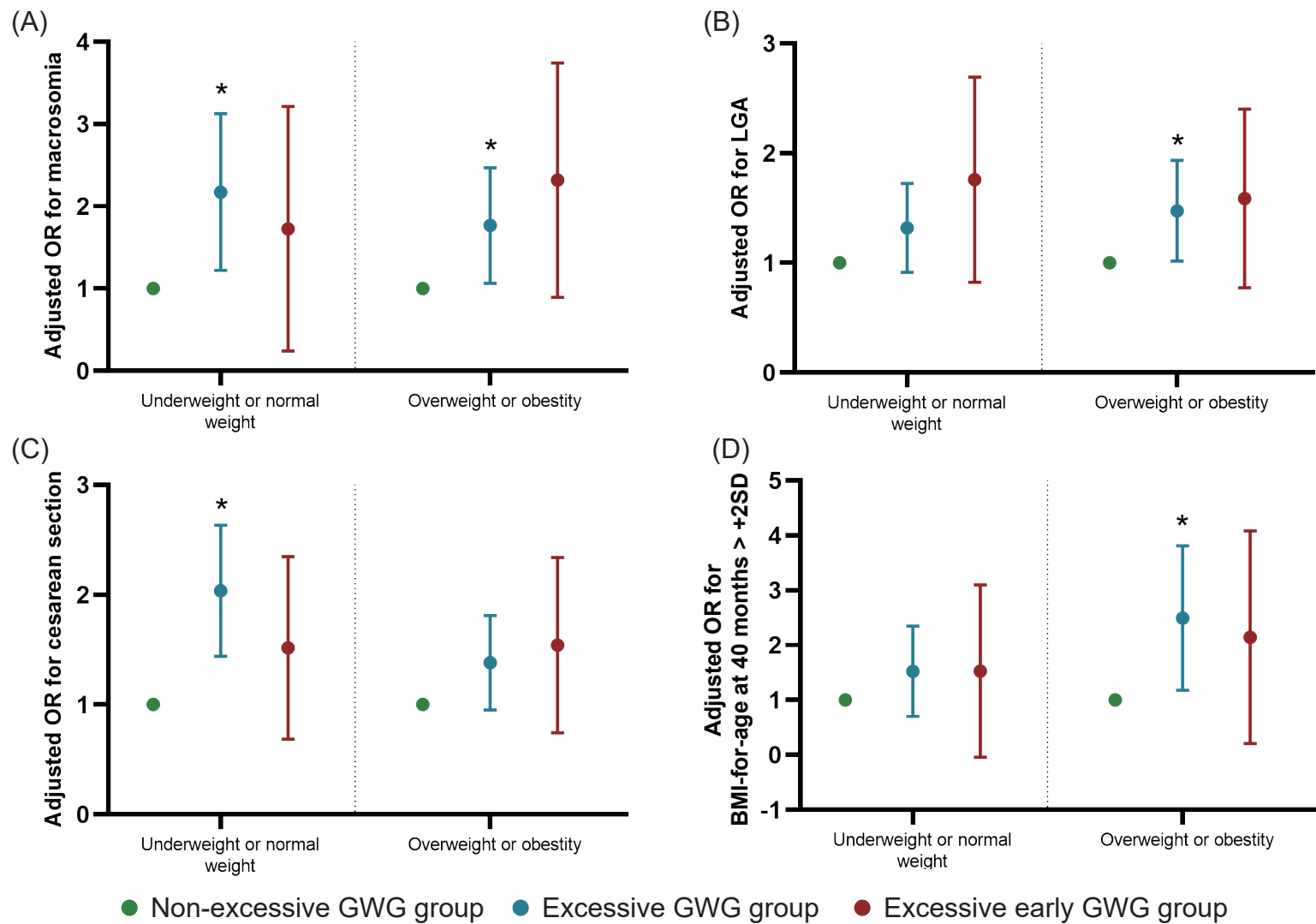

Supplement: Supplementary file 2 — Additional file 2: Figure S2. Adjusted OR for the adverse offspring outcomes in women with different GWG trajectories stratified by body mass index status. OR was adjusted for maternal age, parity, pre-pregnancy BMI, maternal height, and blood glucose levels during OGTT. OR for WFA outcomes at 40 months were additionally adjusted for offspring height at 40 months. * indicated significant difference (p < 0.05) compared to the non-excessive GWG group. [file 13098_2023_1239_MOESM2_ESM.pdf]
